# Genome-wide barcoded transposon screen for cancer drug sensitivity in haploid mouse embryonic stem cells

**DOI:** 10.1038/sdata.2017.20

**Published:** 2017-03-01

**Authors:** Stephen J. Pettitt, Dragomir B. Krastev, Helen N. Pemberton, Yari Fontebasso, Jessica Frankum, Farah L. Rehman, Rachel Brough, Feifei Song, Ilirjana Bajrami, Rumana Rafiq, Fredrik Wallberg, Iwanka Kozarewa, Kerry Fenwick, Javier Armisen-Garrido, Amanda Swain, Aditi Gulati, James Campbell, Alan Ashworth, Christopher J. Lord

**Affiliations:** 1 The CRUK Gene Function Laboratory, The Institute of Cancer Research, Fulham Road, London SW3 6JB, UK; 2 Breast Cancer Now Research Centre, The Institute of Cancer Research, Fulham Road, London SW3 6JB, UK; 3 Tumour Profiling Unit, The Institute of Cancer Research, Fulham Road, London SW3 6JB, UK

**Keywords:** High-throughput screening, Pharmacogenomics, Mutagenesis, Targeted therapies

## Abstract

We describe a screen for cellular response to drugs that makes use of haploid embryonic stem cells. We generated ten libraries of mutants with piggyBac gene trap transposon integrations, totalling approximately 100,000 mutant clones. Random barcode sequences were inserted into the transposon vector to allow the number of cells bearing each insertion to be measured by amplifying and sequencing the barcodes. These barcodes were associated with their integration sites by inverse PCR. We exposed these libraries to commonly used cancer drugs and profiled changes in barcode abundance by Ion Torrent sequencing in order to identify mutations that conferred sensitivity. Drugs tested included conventional chemotherapeutics as well as targeted inhibitors of topoisomerases, poly(ADP-ribose) polymerase (PARP), Hsp90 and WEE1.

## Background & Summary

Haploid mammalian cells only contain one allele of each gene. This property greatly facilitates forward genetic screens, as mutations that would be recessive in a diploid background can directly result in a phenotype. Two types of mammalian haploid cell lines have been used for genetic screens: those derived from a near-haploid human leukaemia^1,2^ and haploid embryonic stem cells derived from activated oocytes^3,4^. Both of these cell types have previously been used for forward genetic screens using insertional mutagens such as transposons and retroviruses. The screens have mainly looked for mutants with selectable phenotypes, either directly (e.g., drug or pathogen resistance^1–3,5^) or via a suitable reporter^6^, although a screen for essential genes has been recently published^7^. We sought to extend these screening systems to look for drug sensitivity phenotypes.

**Figure 1:**
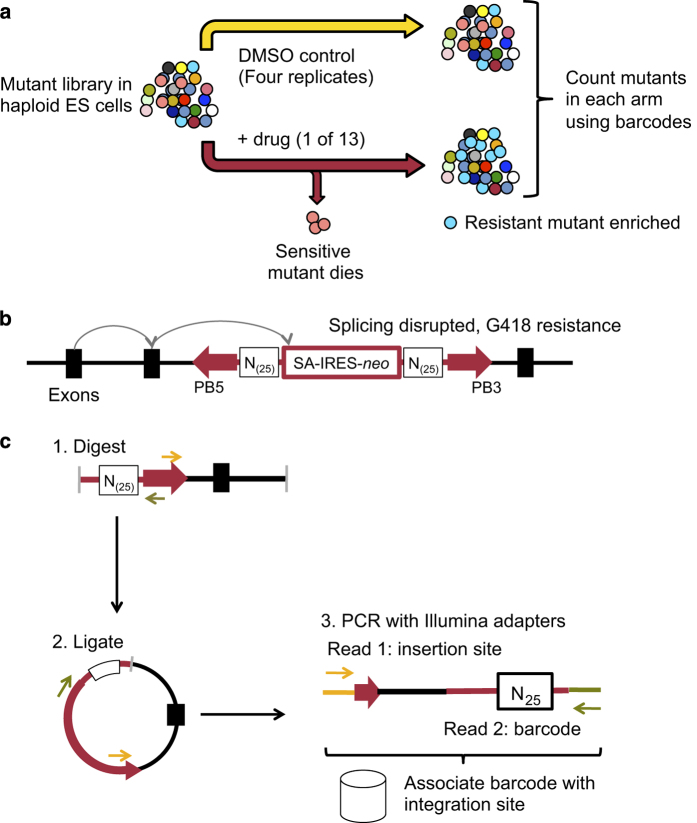
Mutagenesis and screening strategy. (**a**) Screen setup. (**b**) Structure of mutagenic transposon, showing gene trap splice acceptor construct and random barcode regions (N_25_). (**c**) Inverse PCR strategy to associate barcodes with integration sites.

Doing so requires either creating a large array of individual mutants or a system for monitoring the abundance of each mutant in a mixed pool (Fig. 1a). The latter strategy has been used extensively to screen cells with stably-integrated short hairpin RNA (shRNA) expression vectors, by sequencing the distinct shRNA target sequences by next-generation sequencing approaches. This yields read counts for each shRNA that are proportional to the number of cells with integrations of that vector^8^.

To allow us to apply a similar approach to transposon-mutagenised haploid cells, we inserted a random 25 base pair barcode at each end of the transposon, and prepared a complex pool of transposon donor plasmids with different barcodes (Fig. 1b). Since the barcodes are of identical length and average base composition, they can be quantified accurately using a PCR and sequencing approach, allowing the relative number of cells with each barcode to be inferred and compared between samples. However, unlike the hairpin sequence in shRNA vectors, the barcode sequence is of no biological relevance and does not directly identify the disrupted gene in its associated mutant. We used inverse PCR to link barcodes to the disrupted genes that they represent (Fig. 1c). The inverse PCR products generated in this way have the random barcode sequence at one end and the transposon-genome junction at the other. These PCR amplicons were sequenced using a paired end strategy on an Illumina HiSeq 2000 and used to build a database that links barcode sequences to integration sites.

Mutants were generated by transfecting purified haploid embryonic stem cells with transposon and transposase under conditions that generate mainly single copy integrations. The resulting colonies were counted and the equivalent of 1,000 colonies pooled. These 1 k pools were then mixed to generate ten pools of approximately 10,000 mutants each. We carried out screens by exposing these 10 k pools to one of 13 drugs (Table 1) at a concentration designed to kill 50% of wild type cells over the screening period (eight days). Pools treated with DMSO (the drug vehicle) were maintained in parallel to control for differences in mutant growth rates that were not linked to drug exposure. After the drug exposure period we prepared genomic DNA from surviving cells and amplified the barcode sequences. These PCR products were sequenced using the Ion Proton platform, generating read counts that represented the abundance of each barcode. These were merged with the inverse PCR data to produce read counts for each integration site under the different treatment conditions. The edgeR R package^9^ was used to normalise the read counts and generate fold changes and *P*-values for differences in barcode frequency between drug exposed and DMSO exposed cells. Analysis methods are provided to query the data for multiple insertion sites affecting the same gene that are significantly enriched or depleted.

## Methods

### Vector construction

The transposon vector was assembled using InFusion cloning (for sequence see Supplementary Data). Random oligonucleotides were purchased from IDT with appropriate overhangs, and introduced into the vector by InFusion cloning. The cloning reaction was used to transform *E. coli* cells (over 2×10^6^ transformed colonies produced), which were grown overnight in liquid culture with ampicillin and plasmids prepared (Qiagen maxi prep). A previously described hyperactive piggyBac transposase plasmid was used^10^.

### Embryonic stem cell culture and transfection

Haploid mouse embryonic stem cells from 129 strain mice (H129.2) were obtained from the laboratory of Anton Wutz^11^. Cells were cultured as previously described^12^, using 2i+LIF medium up until the point of mutagenesis and conventional ES cell medium with 15% serum and LIF thereafter. Prior to transfection with the transposon plasmids, cells were purified by FACS based on forward and side scatter to enrich for haploid cells^13^. Cells were transfected with limiting amounts of transposon plasmid to obtain mainly single-copy integrations as previously described^14^.

### Mapping of transposon integrations by inverse PCR

Inverse PCR was based on the previously described TRIP protocol^15^. Genomic DNA was prepared from 1 k mutant libraries and five micrograms digested separately with *Bfu*CI (both transposon ends) and either *Hha*I (PB3 end) or *Rsa*I (PB5 end). Digested DNA was diluted to 4.5 ml in T4 ligase buffer and ligated at 16 °C overnight using 800 units of T4 DNA ligase (NEB). Ligated DNA was precipitated by adding 0.1 volume 3 M sodium acetate and two volumes ethanol and centrifuging at 4,700 r.p.m. for 45 min. The DNA pellet was washed once with 70% ethanol and resuspended in 250 μl 5 mM Tris-HCl, pH 8. Primary PCR was carried out using Q5 polymerase (NEB) in a 50 μl reaction volume using 20 μl ligated DNA. Primer sequences and cycling conditions can be found in Supplementary Data. Samples were paired-end sequenced using a HiSeq 2000 instrument, with dark cycles to ignore the low-complexity PCR primer sequences. Barcodes were annotated and the genomic insertion site sequence extracted from the paired reads (*inv-hiseq.rb*). Insertion sites were mapped to the mouse genome (GRCm38) using bwa^16^ (implemented in map.rake) and associated gene information extracted using the ruby Ensembl API (https://github.com/jandot/ruby-ensembl-api, implemented in getgene.rb). Integration sites with fewer than 10 reads/million were discarded, and sites from the same library mapping to sites within 5 bp of each other were combined.

### Barcode recovery and screen analysis

Barcodes were recovered by PCR with primers tailed with barcoded Ion Torrent sequencing adapters (Supplementary Data). The primers used were based on previously published sequences for shRNA vectors^8,17^. PCR reactions were run as previously described^8^ using a total of 30 μg genomic DNA (approximately 1,000 haploid genome equivalents per mutant) template in 38 PCR reactions per sample. Samples were gel-purified at room temperature as previously described^18^ and sequenced on Ion Torrent P1 chips (Life Technologies), loading up to 16 samples per chip in equal amounts. Samples from the same original library were sequenced on the same chip where possible. Read counts were generated for each barcode by parsing FASTQ files (implemented in *barcode-pipe.rake*). Barcodes with high similarity are likely to be the result of sequencing or PCR errors (this was supported by comparison with inverse PCR data) and were grouped and counted together (*group.r*). Fold changes and *P*-values (exact test based on negative binomial distribution) were calculated using the edgeR package^9^ and combined with barcode mapping data (implemented in script *gg_analysis.r*).

### Validation

A combined Cas9-sgRNA lentiviral vector was made by cloning a double stranded oligonucleotide with appropriate overhangs (Supplementary Data) into pLentiCRISPR^19^ (Addgene 49535). JM8A3 ES cells^20^ were transduced, selected in puromycin and colonies picked and screened by PCR and Western blot to identify *Ewsr1* mutants. MCF7 breast cancer cells were transfected with shRNA vectors as previously described^21^. Cell survival after drug exposure was determined using CellTiterGlo (Promega).

### Code availability

Code used to generate and analyse data is deposited on github^22^.

## Data Records

### Mappings

Inverse PCR data was processed as above to generate* hashup.gene* files containing mapping information (Data Citation 1).

### Barcode counts

Fastq files from barcode sequencing experiments were processed as above to generate *tsv* files for each 10 k library screen (Data Citation 2).

### Inverse PCR product sequences

Fastq files containing unmapped inverse PCR products were filtered to exclude truncated reads and reads that did not start with the expected sequence to form* fastqinv* files (Data Citation 3).

## Technical Validation

### Confirmation of mutagenic activity of barcoded transposon

In a preliminary experiment to verify that the new transposon construct was mutagenic, we transfected haploid mouse embryonic stem cells with the barcoded gene trap vector and selected cells with stable integrations using G418 to create a test library. Cells with mutations in components of the mismatch repair pathway are resistant to the modified nucleotide 6-thioguanine (6-TG). Therefore we selected this test library in 2 μM 6-TG. Eighty surviving colonies were isolated. The transposon integrations were mapped by Splinkerette PCR^23^. In 43 clones with mappings, a known mismatch repair gene was disrupted (Table 2 and Supplementary Table 1). Furthermore, the entire canonical mismatch repair pathway was recovered (*Msh2, Msh6, Mlh1, Pms2*), with multiple insertions in *Msh2* and *Msh6* demonstrating effective genome-scale mutagenesis.

### Coverage

**Figure 2:**
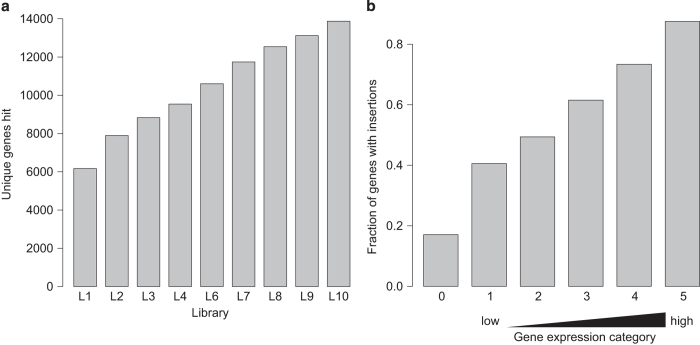
Genomic coverage of libraries. (**a**) Cumulative number of unique genes disrupted in libraries 1–10. (**b**) Breakdown of genes disrupted by expression in mouse embryonic stem cells, The fraction of genes in each category with at least one integration is plotted. Single cell RNA-seq expression data and categorisation have been previously published^28^.

Analysis of the full set of integrations revealed that the libraries approached saturation with 14,132 genes in total affected by a transposon insertion (Fig. 2a). As expected, integrations were biased towards genes that are highly expressed in mouse embryonic stem cells (Fig. 2b).

### Reproducibility

**Figure 3:**
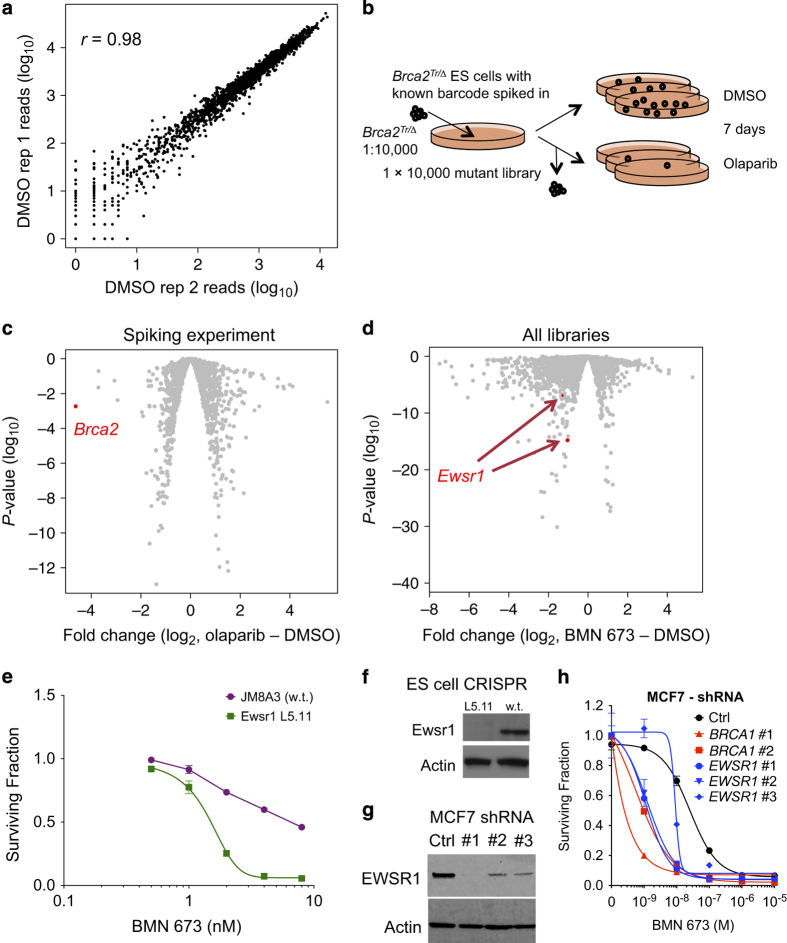
Technical validation. (**a**) Concordance of read counts in two replicate DMSO-treated samples. (**b**) Schematic of spiking experiment. (**c**) Volcano plot of spiking experiment results. *P* values (exact test) and fold changes were calculated using edgeR. The point corresponding to the barcode in the spiked *Brca2* mutant is labelled. (**d**) Volcano plot of barcodes corresponding to *Ewsr1* integrations in all PB3 libraries exposed to the PARP inhibitor BMN 673. (**e**) Validation of *Ewsr1* hit. CRISPR knockout ES cells (L5.11) are more sensitive to BMN 673 than parental JM8A3 cells. Surviving fraction is measured using CellTiterGlo after five days growth in the indicated drug concentration; mean and s.d. of five replicates is plotted. (**f**) Western blot demonstrating knockout of Ewsr1 in CRISPR mutant. (**g**,**h**) Knockdown of the human homologue, *EWSR1,* in MCF7 cells using shRNA causes BMN 673 sensitivity.

Per-barcode read counts were highly correlated in the DMSO-exposed replicates for each library (mean pairwise *r*=0.96, Fig. 3a).

### Proof-of-concept

*Brca2* mutant cells are highly sensitive to the PARP inhibitor olaparib (Lynparza)^24^. ES cells with a targeted mutation in *Brca2* have previously been described^25^. We tagged this cell line by transfecting it with a clonal transposon plasmid with a known barcode. These tagged cells were mixed with one of the 10,000-mutant libraries (library 2) at a 1:10,000 ratio (200 cells to 2 million library cells) to form a spiked library (L2-spk, Fig. 3b). Exposure of this spiked library to olaparib as described above revealed that the tagged barcode was the most depleted in the olaparib-treated population (log_2_ fold change=3.6, *P*<1.4×10^−3^, exact test; Fig. 3c).

### Identification of novel determinants of sensitivity to the PARP inhibitor BMN 673

We asked whether known determinants of PARP inhibitor sensitivity were hits in our screen. The most robust of these, the homologous recombination regulators *Brca1* and *Brca2,* are known to cause a fitness defect in ES cells when fully disrupted, in contrast to the specific truncation allele in the spiked ES cells above, so may not be seen in our screen. Out of a total of 192,631 insertions mapped for the PB3 end, only five were observed in *Brca1* and only one in *Brca2*; these genes have also been shown to be essential in a recent ES cell CRISPR screen^26^.

We therefore looked for novel determinants of PARP inhibitor sensitivity. We applied fold change and *P-*value cutoffs to the analysed barcode abundances for the potent PARP inhibitor BMN 673 (also known as talazoparib^27^) and DMSO exposed libraries. We linked significantly depleted barcodes to their integration sites using the inverse PCR data and observed that multiple barcodes associated with *Ewsr1* integrations were depleted in the samples treated with BMN 673 (Fig. 3d). To confirm this result, we used CRISPR-Cas9 technology to knock out *Ewsr1* in diploid ES cells and verified that these cells were more sensitive to BMN 673 than the parental cell line (4.2-fold lower SF_50_, *P*<0.0001, ANOVA; Fig. 3e,f). This was further validated using three different shRNA constructs to knockdown the human homologue, *EWSR1*, in MCF7 breast cancer cells (20-fold lower SF_50_, *P<*0.001, ANOVA; Fig. 3g,h), suggesting that *EWSR1* might be a genetic determinant of PARP inhibitor sensitivity.

## Usage Notes

Although 1,000 colony equivalents were pooled to form each sublibrary, there are approximately 2,000 integrations identified for each. This could be due to further transposon mobilisation events occurring in colonies after the first cell division, or a subset of cells with stable transposase expression that continue to generate integrations. Many barcodes map to multiple integration sites—this is likely due to lower than expected complexity of the barcoded plasmid population. Transposon ends PB5 and PB3 are referred to in analysis files as Sims and West respectively, corresponding to primer sequences used to prepare sequencing products^8,17^. Mappings were generated for all PB3 (West) libraries and PB5 (Sims) libraries L1 and L2.

## Additional Information

**How to cite this article:** Pettitt, S. J. *et al.* Genome-wide barcoded transposon screen for cancer drug sensitivity in haploid mouse embryonic stem cells. *Sci. Data* 4:170020 doi: 10.1038/sdata.2017.20 (2017).

**Publisher’s note:** Springer Nature remains neutral with regard to jurisdictional claims in published maps and institutional affiliations.

## Supplementary Material



Supplementary Information

## Figures and Tables

**Table 1:** Drugs used in screen.

**Drug**	**Target**	**Libraries screened**
Camptothecin	Topoisomerase I	1, 6, 7, 8, 9, 10
BIIB021	HSP90	1, 2, 3, 6, 7, 8, 9, 10, 2sims
AT13387	HSP90	1, 6, 7, 8, 9, 10
Bleomycin	Double strand breaks	1, 6, 7, 8, 10
Methotrexate	DHFR	1, 2, 3, 7, 8, 9, 10, 2sims
JQ1	BRD4	1, 2, 3, 4, 6, 7, 8, 9, 2sims
KW2478	HSP90	1, 6, 7, 9, 10
BMN 673	PARP1/2	1, 2, 3, 4, 6, 7, 8, 10, 2sims
MK1775	WEE1	1
17-AAG	HSP90	1, 7, 8, 9, 10
Etoposide	Topoisomerase II	1, 6, 7, 10
Decitabine	DNA methyltransferase	1, 6, 7, 8, 9, 10
PF04691502	HSP90	1, 8, 9, 10

**Table 2:** Mutants resistant to 6-thioguanine (6-TG) isolated from haploid cells with barcoded transposon integrations.

**Gene**	**Number of 6-TG resistant colonies**	**Number of insertion sites**
*Msh6*	33	14
*Msh2*	8	4
*Mlh1*	1	1
*Pms2*	1	1
*Csmd1*	2	1
*Eif1a*	2	1
No gene annotated	2	2
Other	29	1
Known mismatch repair genes and others disrupted in more than one colony are shown. For more detail see Supplementary Data.		
