# Supplementary material for: Genome-wide barcoded transposon screen for cancer drug sensitivity in haploid mouse embryonic stem cells
Source: Sci Data. 2017 Mar 1;4:170020. doi: 10.1038/sdata.2017.20 (PMC5332012; doi:10.1038/sdata.2017.20)
Supplement: Supplementary Information [file sdata201720-s2.pdf]

Supplementary Data for Pettitt S.J. *et al* "Genome-wide barcoded transposon screen for cancer drug sensitivity in haploid mouse embryonic stem cells"

Contents:

1 Supplementary Table 1 – 6-TG resistant mutants
2 PCR conditions
3–4 Oligonucleotide sequences
5–10 Barcoded transposon plasmid sequence in GenBank format

Supplementary Table 1: Full Sanger sequencing mapping data for 6-TG resistant colonies

Note that transfection conditions were not optimised for single-copy insertions in this screen, therefore there are multiple insertions for many clones.

Colonies	PB5 chr	PB5 pos	PB3 chr	PB3 pos	PB5 gene	PB3 gene
**13**	**17**	**87,980,842**	**17**	**87,980,841**	***Msh6***	
**5**	**17**	**14,075,902**	**17**	**87,976,095**		***Msh6***
**4**	**17**	**87,981,979**	**17**	**87,981,978**	***Msh6***	
**4**	**NA**	**NA**	**17**	**87,673,130**	***NA***	***Msh2***
2	8	15,983,713	2	30,885,944	*Csmd1*	
2	18	46,607,977	7	72,326,037	*Eif1a*	
**2**	**17**	**87,695,676**	**NA**	**NA**	***Msh2***	***NA***
2	2	66,181,060	2	66,181,064		
2	13	80,932,417	3	7,641,626		
**1**	**17**	**87,696,147**	**17**	**87,696,154**	***Msh2***	
**1**	**17**	**87,686,487**	**NA**	**NA**	***Msh2***	***NA***
**1**	**17**	**87,977,132**	**11**	**11,871,662**	***Msh6***	
**1**	**17**	**87,976,095**	**17**	**30,602,248**	***Msh6***	
**1**	**17**	**87,986,894**	**17**	**87,986,950**	***Msh6***	
**1**	**17**	**87,985,785**	**17**	**87,985,784**	***Msh6***	
**1**	**17**	**87,986,027**	**19**	**47,789,915**	***Msh6***	
**1**	**17**	**87,977,320**	**2**	**166,481,574**	***Msh6***	
**1**	**5**	**143,913,337**	**NA**	**NA**	***Pms2***	***NA***
**1**	**17**	**87,975,970**	**12**	**27,420,386**	***Msh6***	
**1**	**NA**	**NA**	**9**	**111,270,757**	***NA***	***Mlh1***
**1**	**NA**	**NA**	**17**	**87,976,106**	***NA***	***Msh6***
**1**	**NA**	**NA**	**17**	**87,982,198**	***NA***	***Msh6***
**1**	**NA**	**NA**	**17**	**87,982,154**	***NA***	***Msh6***
**1**	**NA**	**NA**	**17**	**87,986,026**	***NA***	***Msh6***
1	12	105,216,775	17	87,708,692	*Tcl1*	
1	2	4,575,487	17	87,976,094	*Frmd4a*	
1	8	24,626,040	11	107,448,585	*Adam18*	
1	2	91,018,524	15	53,929,745	*Celf1*	
1	4	125,610,100	3	41,607,029	*Grik3*	
1	2	155,202,987	NA	NA	*Itch*	*NA*
1	10	42,394,530	9	57,048,835	*Lace1*	
1	5	45,495,981	8	71,239,563	*Lap3*	
1	NA	NA	X	144,362,807	*NA*	*Alg13*
1	NA	NA	17	16,702,867	*NA*	*NA*
1	NA	NA	4	4,133,834	*NA*	*Penk*
1	NA	NA	1	189,730,157	*NA*	*Ptpn14*
1	NA	NA	14	63,494,809	*NA*	*Tdh*
1	NA	NA	5	116,076,026	*NA*	*Tmem233*
1	NA	NA	X	8,433,292	*NA*	
1	NA	NA	14	14,314,940	*NA*	
1	NA	NA	6	112,537,455	*NA*	
1	NA	NA	17	78,948,231	*NA*	
1	NA	NA	10	82,175,718	*NA*	
1	15	81,397,057	NA	NA	*St13*	*NA*
1	5	116,076,073	16	33,405,964	*Tmem233*	
1	19	26,828,382	8	94,081,687		*Bbs2*
1	15	84,191,907	NA	NA		*NA*
1	3	38,420,211	NA	NA		*NA*
1	10	30,193,691	NA	NA		*NA*
1	2	118,176,835	4	121,199,306		*Rlf*
1	15	93,632,521	2	85,902,464		
1	6	49,236,495	19	43,920,226		
1	13	94,902,797	13	94,903,088		

Supplementary Data - PCR conditions

Barcode PCR

Temperature (°C)	Time (s)	Cycles
98	30	
98	10	
60	10	25 cycles
72	10	
72	60	

Inverse PCR (Primary and secondary)

Temperature (°C)	Time (s)	Cycles
98	30	
98	10	
65	10	18 cycles
72	30	
72	60	

Indexing PCR

Temperature (°C)	Time (s)	Cycles
98	30	
51.7	30	
72	30	6 cycles
72	5	

Oligonucleotide sequences

Name	Sequence	Purpose
Sims_ion_1	CCATCTCATCCCTGCGTGTCTCCGACTCAGCTAAGGTAACgatCTAAAGTAGCCCCTTGAATTC	Barcode amplification for Ion Torrent
Sims_ion_2	CCATCTCATCCCTGCGTGTCTCCGACTCAGTAAGGAGAACgatCTAAAGTAGCCCCTTGAATTC	Barcode amplification for Ion Torrent
Sims_ion_3	CCATCTCATCCCTGCGTGTCTCCGACTCAGAAGAGGATTCgatCTAAAGTAGCCCCTTGAATTC	Barcode amplification for Ion Torrent
Sims_ion_4	CCATCTCATCCCTGCGTGTCTCCGACTCAGTACCAAGATCgatCTAAAGTAGCCCCTTGAATTC	Barcode amplification for Ion Torrent
Sims_ion_5	CCATCTCATCCCTGCGTGTCTCCGACTCAGCAGAAGGAACgatCTAAAGTAGCCCCTTGAATTC	Barcode amplification for Ion Torrent
Sims_ion_6	CCATCTCATCCCTGCGTGTCTCCGACTCAGCTGCAAGTTCgatCTAAAGTAGCCCCTTGAATTC	Barcode amplification for Ion Torrent
Sims_ion_7	CCATCTCATCCCTGCGTGTCTCCGACTCAGTTCGTGATTCgatCTAAAGTAGCCCCTTGAATTC	Barcode amplification for Ion Torrent
Sims_ion_8	CCATCTCATCCCTGCGTGTCTCCGACTCAGTTCCGATAACgatCTAAAGTAGCCCCTTGAATTC	Barcode amplification for Ion Torrent
Sims_ion_9	CCATCTCATCCCTGCGTGTCTCCGACTCAGTGAGCGGAACgatCTAAAGTAGCCCCTTGAATTC	Barcode amplification for Ion Torrent
Sims_ion_10	CCATCTCATCCCTGCGTGTCTCCGACTCAGCTGACCGAACgatCTAAAGTAGCCCCTTGAATTC	Barcode amplification for Ion Torrent
Sims_ion_11	CCATCTCATCCCTGCGTGTCTCCGACTCAGTCCTCGAATCgatCTAAAGTAGCCCCTTGAATTC	Barcode amplification for Ion Torrent
Sims_ion_12	CCATCTCATCCCTGCGTGTCTCCGACTCAGTAGGTGGTTCgatCTAAAGTAGCCCCTTGAATTC	Barcode amplification for Ion Torrent
Sims_ion_13	CCATCTCATCCCTGCGTGTCTCCGACTCAGTCTAACGGACgatCTAAAGTAGCCCCTTGAATTC	Barcode amplification for Ion Torrent
Sims_ion_14	CCATCTCATCCCTGCGTGTCTCCGACTCAGTTGGAGTGTCgatCTAAAGTAGCCCCTTGAATTC	Barcode amplification for Ion Torrent
Sims_ion_15	CCATCTCATCCCTGCGTGTCTCCGACTCAGTCTAGAGGTCgatCTAAAGTAGCCCCTTGAATTC	Barcode amplification for Ion Torrent
Sims_ion_16	CCATCTCATCCCTGCGTGTCTCCGACTCAGTCTGGATGACgatCTAAAGTAGCCCCTTGAATTC	Barcode amplification for Ion Torrent
West_ion_1	CCATCTCATCCCTGCGTGTCTCCGACTCAGCTAAGGTAACgatCTAAAGCGCATGCTCCAGACTG	Barcode amplification for Ion Torrent
West_ion_2	CCATCTCATCCCTGCGTGTCTCCGACTCAGTAAGGAGAACgatCTAAAGCGCATGCTCCAGACTG	Barcode amplification for Ion Torrent
West_ion_3	CCATCTCATCCCTGCGTGTCTCCGACTCAGAAGAGGATTCgatCTAAAGCGCATGCTCCAGACTG	Barcode amplification for Ion Torrent
West_ion_4	CCATCTCATCCCTGCGTGTCTCCGACTCAGTACCAAGATCgatCTAAAGCGCATGCTCCAGACTG	Barcode amplification for Ion Torrent
West_ion_5	CCATCTCATCCCTGCGTGTCTCCGACTCAGCAGAAGGAACgatCTAAAGCGCATGCTCCAGACTG	Barcode amplification for Ion Torrent
West_ion_6	CCATCTCATCCCTGCGTGTCTCCGACTCAGCTGCAAGTTCgatCTAAAGCGCATGCTCCAGACTG	Barcode amplification for Ion Torrent
West_ion_7	CCATCTCATCCCTGCGTGTCTCCGACTCAGTTCGTGATTCgatCTAAAGCGCATGCTCCAGACTG	Barcode amplification for Ion Torrent
West_ion_8	CCATCTCATCCCTGCGTGTCTCCGACTCAGTTCCGATAACgatCTAAAGCGCATGCTCCAGACTG	Barcode amplification for Ion Torrent
West_ion_9	CCATCTCATCCCTGCGTGTCTCCGACTCAGTGAGCGGAACgatCTAAAGCGCATGCTCCAGACTG	Barcode amplification for Ion Torrent
West_ion_10	CCATCTCATCCCTGCGTGTCTCCGACTCAGCTGACCGAACgatCTAAAGCGCATGCTCCAGACTG	Barcode amplification for Ion Torrent
West_ion_11	CCATCTCATCCCTGCGTGTCTCCGACTCAGTCCTCGAATCgatCTAAAGCGCATGCTCCAGACTG	Barcode amplification for Ion Torrent
West_ion_12	CCATCTCATCCCTGCGTGTCTCCGACTCAGTAGGTGGTTCgatCTAAAGCGCATGCTCCAGACTG	Barcode amplification for Ion Torrent

West_ion_13	CCATCTCATCCCTGCGTGTCTCCGACTCAGTCTAACGGACgatCTAAAGCGCATGCTCCAGACTG	Barcode amplification for Ion Torrent
West_ion_14	CCATCTCATCCCTGCGTGTCTCCGACTCAGTTGGAGTGTCgatCTAAAGCGCATGCTCCAGACTG	Barcode amplification for Ion Torrent
West_ion_15	CCATCTCATCCCTGCGTGTCTCCGACTCAGTCTAGAGGTCgatCTAAAGCGCATGCTCCAGACTG	Barcode amplification for Ion Torrent
West_ion_16	CCATCTCATCCCTGCGTGTCTCCGACTCAGTCTGGATGACgatCTAAAGCGCATGCTCCAGACTG	Barcode amplification for Ion Torrent
Sims_ion_R	CCTCTCTATGGGCAGTCGGTGATCCTCAGCTAGTGAAGCCACAGATGTA	Barcode amplification for Ion Torrent
West_ion_R	CCTCTCTATGGGCAGTCGGTGATCCTCAGCCTTGAACCTCCTCGTTCGACC	Barcode amplification for Ion Torrent
PB3_inv_R1	CTCTTCTGCACTTGAACCTCCTC	Inverse PCR (first round)
PB3_inv_F1	TTCAAGAATGCATGCGTCAATTTT	Inverse PCR (first round)
PB5_inv_F1	GATATACAGACCGATAAAACACATGCGTCA	Inverse PCR (first round)
PB5_inv_R1	AGTCAGAAACAACTTTGGCCTAAA	Inverse PCR (first round)
PB5_illu_F	ACACTCTTTCCCTACACGACGCTCTTCCGATCTGTACGTCACAATATGATTATCTTTCTAG	Inverse PCR for HiSeq (second round)
PB3_illu_F	ACACTCTTTCCCTACACGACGCTCTTCCGATCTGTCAATTTTACGCAGACTATCTTTCTAG	Inverse PCR for HiSeq (second round)
PB5_illu_R_full	gtgactggagttcAGACGTGTGCTCTTCCGATCtGCCTAAAGTAGCCCCTTGAATTC	Inverse PCR for HiSeq (second round)
PB3_illu_R_full	gtgactggagttcAGACGTGTGCTCTTCCGATCtCTTCTGCACTTGAACCTCCTCGTT	Inverse PCR for HiSeq (second round)
Ewsr1_gRNA_F	CACCgTATGCAGTCTGCCCGTAGG	Ewsr1 CRISPR vector cloning
Ewsr1_gRNA_R	AAACCCTACGGGCAGACTGCATAc	Ewsr1 CRISPR vector cloning
Ewsr1-F	AAGCAGCTCAGTTTAAGAAGGC	Ewsr1 CRISPR genotyping
Ewsr1-R	CAAGGTGCTGGCTGAATCTTG	Ewsr1 CRISPR genotyping

```
LOCUS       pPB_SA_IRES_Neo_        6152 bp ds-DNA     circular     06-AUG-2013
DEFINITION  .
ACCESSION   
VERSION     
SOURCE      .
  ORGANISM  .
COMMENT     
COMMENT     ApEinfo:methylated:0
FEATURES             Location/Qualifiers
     primer_bind     complement(3317..3336)
                     /label=T3
                     /ApEinfo_fwdcolor=cyan
                     /ApEinfo_revcolor=green
                     /ApEinfo_graphicformat=arrow_data {{0 1 2 0 0 -1} {} 0}
                     width 5 offset 0
     misc_feature    2868..3177
                     /label=PB3
                     /ApEinfo_fwdcolor=#0cfeff
                     /ApEinfo_revcolor=#0cfeff
                     /ApEinfo_graphicformat=arrow_data {{} {0 1 2 0 0 -1} 0}
                     width 5 offset 0
     primer_bind     6106..6123
                     /label=M13-fwd
                     /ApEinfo_fwdcolor=cyan
                     /ApEinfo_revcolor=green
                     /ApEinfo_graphicformat=arrow_data {{0 1 2 0 0 -1} {} 0}
                     width 5 offset 0
     misc_feature    530..966
                     /note="the human bcl-2 gene splice acceptor (intron 2 -
                     modified exon 3)"
                     /label=SA
                     /ApEinfo_fwdcolor=#ff80ed
                     /ApEinfo_revcolor=#ffcc66
                     /ApEinfo_graphicformat=arrow_data {{0 1 2 0 0 -1} {} 0}
                     width 5 offset 0
     primer_bind     complement(3354..3374)
                     /label=M13-rev
                     /ApEinfo_fwdcolor=cyan
                     /ApEinfo_revcolor=green
                     /ApEinfo_graphicformat=arrow_data {{0 1 2 0 0 -1} {} 0}
                     width 5 offset 0
     misc_feature    58..320
                     /label=PB5
                     /ApEinfo_fwdcolor=cyan
                     /ApEinfo_revcolor=green
                     /ApEinfo_graphicformat=arrow_data {{0 1 2 0 0 -1} {} 0}
                     width 5 offset 0
     primer_bind     6132..6152
                     /label=T7
                     /ApEinfo_fwdcolor=cyan
                     /ApEinfo_revcolor=green
                     /ApEinfo_graphicformat=arrow_data {{0 1 2 0 0 -1} {} 0}
                     width 5 offset 0
     promoter        1573..1610
                     /label=gb3
                     /ApEinfo_fwdcolor=#1014ff
                     /ApEinfo_revcolor=green
                     /ApEinfo_graphicformat=arrow_data {{0 1 2 0 0 -1} {} 0}
                     width 5 offset 0
     rep_origin      complement(3742..4424)
```

```
                 /label=ColE1 origin
                 /ApEinfo_fwdcolor=gray50
                 /ApEinfo_revcolor=gray50
                 /ApEinfo_graphicformat=arrow_data {{0 1 2 0 0 -1} {} 0}
                 width 5 offset 0
misc_feature     2799..2821
                 /label=primer-right
                 /ApEinfo_fwdcolor=#ff0006
                 /ApEinfo_revcolor=green
                 /ApEinfo_graphicformat=arrow_data {{0 1 2 0 0 -1} {} 0}
                 width 5 offset 0
rep_origin       complement(5642..5948)
                 /label=F1 ori
                 /ApEinfo_fwdcolor=gray50
                 /ApEinfo_revcolor=gray50
                 /ApEinfo_graphicformat=arrow_data {{0 1 2 0 0 -1} {} 0}
                 width 5 offset 0
misc_feature     785..787
                 /note="an artificial stop codon"
                 /label=an artificial stop codon
                 /ApEinfo_fwdcolor=#ff8000
                 /ApEinfo_revcolor=#ffcc66
                 /ApEinfo_graphicformat=arrow_data {{0 1 2 0 0 -1} {} 0}
                 width 5 offset 0
CDS              complement(5967..6035)
                 /label=LacZ alpha
                 /ApEinfo_fwdcolor=#6495ed
                 /ApEinfo_revcolor=#6495ed
                 /ApEinfo_graphicformat=arrow_data {{0 1 2 0 0 -1} {} 0}
                 width 5 offset 0
misc_feature     416..437
                 /label=primer-Left
                 /ApEinfo_fwdcolor=#ff0612
                 /ApEinfo_revcolor=green
                 /ApEinfo_graphicformat=arrow_data {{} {0 1 2 0 0 -1} 0}
                 width 5 offset 0
misc_binding     complement(3380..3402)
                 /label=LacO
                 /ApEinfo_fwdcolor=#6495ed
                 /ApEinfo_revcolor=#6495ed
                 /ApEinfo_graphicformat=arrow_data {{0 1 2 0 0 -1} {} 0}
                 width 5 offset 0
promoter         980..1559
                 /label=ires
                 /ApEinfo_fwdcolor=#ff970a
                 /ApEinfo_revcolor=green
                 /ApEinfo_graphicformat=arrow_data {{0 1 2 0 0 -1} {} 0}
                 width 5 offset 0
CDS              complement(4522..5181)
                 /label=AmpR
                 /ApEinfo_fwdcolor=yellow
                 /ApEinfo_revcolor=yellow
                 /ApEinfo_graphicformat=arrow_data {{0 1 2 0 0 -1} {} 0}
                 width 5 offset 0
misc_feature     438..458
                 /label=p5+mir3
                 /ApEinfo_fwdcolor=#32ff82
                 /ApEinfo_revcolor=green
                 /ApEinfo_graphicformat=arrow_data {{0 1 2 0 0 -1} {} 0}
                 width 5 offset 0
```

```
CDS             1611..2457
                /label=neo
                /ApEinfo_fwdcolor=cyan
                /ApEinfo_revcolor=green
                /ApEinfo_graphicformat=arrow_data {{0 1 2 0 0 -1} {} 0}
                width 5 offset 0
misc_feature    484..502
                /label=p7+Loop
                /ApEinfo_fwdcolor=#07ffa6
                /ApEinfo_revcolor=green
                /ApEinfo_graphicformat=arrow_data {{} {0 1 2 0 0 -1} 0}
                width 5 offset 0
polyA_site      2580..2701
                /label=SV40 polyA
                /ApEinfo_fwdcolor=#0fff34
                /ApEinfo_revcolor=green
                /ApEinfo_graphicformat=arrow_data {{0 1 2 0 0 -1} {} 0}
                width 5 offset 0
misc_feature    2731..2752
                /label=PB3seqF
                /ApEinfo_fwdcolor=#00ff4d
                /ApEinfo_revcolor=green
                /ApEinfo_graphicformat=arrow_data {{0 1 2 0 0 -1} {} 0}
                width 5 offset 0
misc_feature    2778..2798
                /label=PB3seq_R
                /ApEinfo_fwdcolor=#11ff66
                /ApEinfo_revcolor=green
                /ApEinfo_graphicformat=arrow_data {{} {0 1 2 0 0 -1} 0}
                width 5 offset 0
primer_bind     2898..2925
                /label=PB3_seq_R
                /ApEinfo_fwdcolor=#ffe609
                /ApEinfo_revcolor=green
                /ApEinfo_graphicformat=arrow_data {{} {0 1 2 0 0 -1} 0}
                width 5 offset 0
misc_feature    459..483
                /label=PB5 barcode
                /ApEinfo_fwdcolor=#f111ff
                /ApEinfo_revcolor=green
                /ApEinfo_graphicformat=arrow_data {{} {} 0} width 5 offset
                0
misc_feature    2753..2777
                /label=PB3 barcode
                /ApEinfo_fwdcolor=#dc1dff
                /ApEinfo_revcolor=green
                /ApEinfo_graphicformat=arrow_data {{} {} 0} width 5 offset
                0
CDS             1666..2457
                /label=Kan/neoR
                /ApEinfo_fwdcolor=yellow
                /ApEinfo_revcolor=yellow
                /ApEinfo_graphicformat=arrow_data {{0 1 2 0 0 -1} {} 0}
                width 5 offset 0
misc_feature    116..145
                /label=PB5_inv_F1
                /ApEinfo_fwdcolor=cyan
                /ApEinfo_revcolor=green
                /ApEinfo_graphicformat=arrow_data {{0 1 2 0 0 -1} {} 0}
                width 5 offset 0
```

```
     misc_feature    83..110
                     /label=PB5_inv_F2
                     /ApEinfo_fwdcolor=cyan
                     /ApEinfo_revcolor=green
                     /ApEinfo_graphicformat=arrow_data {{0 1 2 0 0 -1} {} 0}
                     width 5 offset 0
     misc_feature    419..442
                     /label=PB5_inv_R1
                     /ApEinfo_fwdcolor=cyan
                     /ApEinfo_revcolor=green
                     /ApEinfo_graphicformat=arrow_data {{0 1 2 0 0 -1} {} 0}
                     width 5 offset 0
     misc_feature    436..458
                     /label=PB5_inv_R2
                     /ApEinfo_fwdcolor=cyan
                     /ApEinfo_revcolor=green
                     /ApEinfo_graphicformat=arrow_data {{0 1 2 0 0 -1} {} 0}
                     width 5 offset 0
     misc_feature    2786..2808
                     /label=PB3_inv_F1
                     /ApEinfo_fwdcolor=cyan
                     /ApEinfo_revcolor=green
                     /ApEinfo_graphicformat=arrow_data {{0 1 2 0 0 -1} {} 0}
                     width 5 offset 0
     misc_feature    3129..3152
                     /label=PB3_inv_F2
                     /ApEinfo_fwdcolor=cyan
                     /ApEinfo_revcolor=green
                     /ApEinfo_graphicformat=arrow_data {{0 1 2 0 0 -1} {} 0}
                     width 5 offset 0
     misc_feature    2783..2806
                     /label=PB3_inv_R1
                     /ApEinfo_fwdcolor=cyan
                     /ApEinfo_revcolor=green
                     /ApEinfo_graphicformat=arrow_data {{0 1 2 0 0 -1} {} 0}
                     width 5 offset 0
     misc_feature    3153..3176
                     /label=PB3_inv_R2
                     /ApEinfo_fwdcolor=cyan
                     /ApEinfo_revcolor=green
                     /ApEinfo_graphicformat=arrow_data {{0 1 2 0 0 -1} {} 0}
                     width 5 offset 0
ORIGIN
        1 CGAATTGGCG GTATTCACGA CAGCAGGCTG AATAATAAAA AAATTAGAAA CTATTATTTA
       61 ACCCTAGAAA GATAATCATA TTGTGACGTA CGTTAAAGAT AATCATGCGT AAAATTGACG
      121 CATGTGTTTT ATCGGTCTGT ATATCGAGGT TTATTTATTA ATTTGAATAG ATATTAAGTT
      181 TTATTATATT TACACTTACA TACTAATAAT AAATTCAACA AACAATTTAT TTATGTTTAT
      241 TTATTTATTA AAAAAAAACA AAAACTCAAA ATTTCTTCTA TAAAGTAACA AAACTTTTAA
      301 ACATTCTCTC TTTTACAAAA ataaacttat tttgtacttt aaaaacagtc atgttgtatt
      361 ataaaataag taattagctt aacctataca taatagaaac aaattatact tattaGTCAG
      421 TCAGAAACAA CTTTGGCCTA AAGTAGCCCC TTGAATTCNN NNNNNNNNNN NNNNNNNNNN
      481 NNNTACATCT GTggcttcac tagctagcgc attagttatt aatccgggat cTGCTGTATC
      541 TCTAAGatgg ctggcacgta aagagggtct caataaatat gccttgaatt aatgaaaaca
      601 gtggtttctg aatcatggcc tccaaagagc atttgccttt tcccctctca cccttctgaa
      661 ggtgcccagg ctgccctggc aggatgcctc tttctctgtg gggtggcatt ctctgctctc
      721 tctctaatac tctttctttt tcttccctcc tctcccccaa ctgcaggatg cctttgtgga
      781 actgtaaggc cccagcatgc ggcctctgtt tgatttctcc tggctgtctc tgaagactct
      841 gctcagtttg gccctggtgg gagcttgcat caccctgggt gcctatctgg gccacaagtg
      901 aagtcaacat gcctgcccca aacaaatatg caaaaggttc aCTAAAGCAG TAGAAATAAT
      961 ATGCATCCGC GGTGGTAAGA ATTCCGCCCC TCTCCCTCCC CCCCCCCTAA CGTTACTGGC
```

1021 CGAAGCCGCT TGGAATAAGG CCGGTGTGCG TTTGTCTATA TGTTATTTTC CACCATATTG
1081 CCGTCTTTTG GCAATGTGAG GGCCCGGAAA CCTGGCCCTG TCTTCTTGAC GAGCATTCCT
1141 AGGGGTCTTT CCCCTCTCGC CAAAGGAATG CAAGGTCTGT TGAATGTCGT GAAGGAAGCA
1201 GTTCCTCTGG AAGCTTCTTG AAGACAAACA ACGTCTGTAG CGACCCTTTG CAGGCAGCGG
1261 AACCCCCCAC CTGGCGACAG GTGCCTCTGC GGCCAAAAGC CACGTGTATA AGATACACCT
1321 GCAAAGGCGG CACAACCCCA GTGCCACGTT GTGAGTTGGA TAGTTGTGGA AAGAGTCAAA
1381 TGGCTCTCCT CAAGCGTATT CAACAAGGGG CTGAAGGATG CCCAGAAGGT ACCCCATTGT
1441 ATGGGATCTG ATCTGGGGCC TCGGTGCACA TGCTTTACAT GTGTTTAGTC GAGGTTAAAA
1501 AACGTCTAGG CCCCCCGAAC CACGGGGACG TGGTTTTCCT TTGAAAAACA CGATGATAAT
1561 ATGGCCACAA CCTGTTACAT TGCACAAGAT AAAAATATAT CATCACGAAC AGTAAAACTG
1621 TCTGCTTACA TAAACAGTAA TACAAGGGGT GTTATgggat cggccattga acaagATGGA
1681 TTGCACGCAG GTTCTCCGGC CGCTTGGGTG GAGAGGCTAT TCGGCTATGA CTGGGCACAA
1741 CAGACAATCG GCTGCTCTGA TGCCGCCGTG TTCCGGCTGT CAGCGCAGGG GCGCCCGGTT
1801 CTTTTTGTCA AGACCGACCT GTCCGGTGCC CTGAATGAAC TGCAGGACGA GGCAGCGCGG
1861 CTATCGTGGC TGGCCACGAC GGGCGTTCCT TGCGCAGCTG TGCTCGACGT TGTCACTGAA
1921 GCGGGAAGGG ACTGGCTGCT ATTGGGCGAA GTGCCGGGGC AGGATCTCCT GTCATCTCAC
1981 CTTGCTCCTG CCGAGAAAGT ATCCATCATG GCTGATGCAA TGCGGCGGCT GCATACGCTT
2041 GATCCGGCTA CCTGCCCATT CGACCACCAA GCGAAACATC GCATCGAGCG AGCACGTACT
2101 CGGATGGAAG CCGGTCTTGT CGATCAGGAT GATCTGGACG AAGAGCATCA GGGGCTCGCG
2161 CCAGCCGAAC TGTTCGCCAG GCTCAAGGCG CGCATGCCCG ACGGCGAGGA TCTCGTCGTG
2221 ACCCATGGCG ATGCCTGCTT GCCGAATATC ATGGTGGAAA ATGGCCGCTT TTCTGGATTC
2281 ATCGACTGTG GCCGGCTGGG TGTGGCGGAC CGCTATCAGG ACATAGCGTT GGCTACCCGT
2341 GATATTGCTG AAGAGCTTGG CGGCGAATGG GCTGACCGCT TCCTCGTGCT TTACGGTATC
2401 GCCGCTCCCG ATTCGCAGCG CATCGCCTTC TATCGCCTTC TTGACGAGTT CTTCTGAAGC
2461 GGCCGCGACT CTAGATCATA ATCAGCCATA CCACATTTGT AGAGGTTTTA CTTGCTTTAA
2521 AAAACCTCCC ACACCTCCCC CTGAACCTGA AACATAAAAT GAATGCAATT GTTGTTGTTA
2581 ACTTGTTTAT TGCAGCTTAT AATGGTTACA AATAAAGCAA TAGCATCACA AATTTCACAA
2641 ATAAAGCATT TTTTTCACTG CATTCTAGTT GTGGTTtgtc caaactcatc aatgtatcTT
2701 AAGGCGTAAA TTGTAAGGGT ACCAGGATCC CTAAAGCGCA TGCTCCAGAC TGNNNNNNNN
2761 NNNNNNNNNN NNNNNNNGGT CGAACGAGGA GGTTCAAGTG CAGAAGAGCA GAGAGGATAT
2821 Gctcatcgtc taaagaacta cccattttat tatatattag tcacgatATC TATAACAAGA
2881 AAATATATAT ATAATAAgtt atcacgtaag tagaacatga aataaCAATA TAATTATCGT
2941 ATGAGTTAAA TCTTAAAAGT CACGTAAAAG ATAATCATGC GTCATTTTGA CTCACGCGGT
3001 CGTTATAGTT CAAAATCAGT GACACTTACC GCATTGACAA GCACGCCTCA CGGGAGCTCC
3061 AAGCGGCGAC TGAGATGTCC TAAATGCACA GCGACGGATT CGCGCTATTT AGAAAGAGAG
3121 AGCAATATTT CAAGAATGCA TGCGTCAATT TTACGCAGAC TATCTTTCTA GGGTTAAAAA
3181 AGATTTGCGC TTTACTCGAC CTAAACTTTA AACACGTCAT AGAATCTTCG TTTGACAAAA
3241 ACCACATTGT GGCCAAGCTG TGTGACGCGA CGCGCGCTAA AGAATGGCAA ACCAAGTCGC
3301 GCGAGCCAGC TTTTGTTCCC TTTAGTGAGG GTTAATTGCG CGCTTGGCGT AATCATGGTC
3361 ATAGCTGTTT CCTGTGTGAA ATTGTTATCC GCTCACAATT CCACACAACA TACGAGCCGG
3421 AAGCATAAAG TGTAAAGCCT GGGGTGCCTA ATGAGTGAGC TAACTCACAT TAATTGCGTT
3481 GCGCTCACTG CCCGCTTTCC AGTCGGGAAA CCTGTCGTGC CAGCTGCATT AATGAATCGG
3541 CCAACGCGCG GGGAGAGGCG GTTTGCGTAT TGGGCGCTCT TCCGCTTCCT CGCTCACTGA
3601 CTCGCTGCGC TCGGTCGTTC GGCTGCGGCG AGCGGTATCA GCTCACTCAA AGGCGGTAAT
3661 ACGGTTATCC ACAGAATCAG GGGATAACGC AGGAAAGAAC ATGTGAGCAA AAGGCCAGCA
3721 AAAGGCCAGG AACCGTAAAA AGGCCGCGTT GCTGGCGTTT TTCCATAGGC TCCGCCCCCC
3781 TGACGAGCAT CACAAAAATC GACGCTCAAG TCAGAGGTGG CGAAACCCGA CAGGACTATA
3841 AAGATACCAG GCGTTTCCCC CTGGAAGCTC CCTCGTGCGC TCTCCTGTTC CGACCCTGCC
3901 GCTTACCGGA TACCTGTCCG CCTTTCTCCC TTCGGGAAGC GTGGCGCTTT CTCATAGCTC
3961 ACGCTGTAGG TATCTCAGTT CGGTGTAGGT CGTTCGCTCC AAGCTGGGCT GTGTGCACGA
4021 ACCCCCCGTT CAGCCCGACC GCTGCGCCTT ATCCGGTAAC TATCGTCTTG AGTCCAACCC
4081 GGTAAGACAC GACTTATCGC CACTGGCAGC AGCCACTGGT AACAGGATTA GCAGAGCGAG
4141 GTATGTAGGC GGTGCTACAG AGTTCTTGAA GTGGTGGCCT AACTACGGCT ACACTAGAAG
4201 GACAGTATTT GGTATCTGCG CTCTGCTGAA GCCAGTTACC TTCGGAAAAA GAGTTGGTAG
4261 CTCTTGATCC GGCAAACAAA CCACCGCTGG TAGCGGTGGT TTTTTTGTTT GCAAGCAGCA
4321 GATTACGCGC AGAAAAAAAG GATCTCAAGA AGATCCTTTG ATCTTTTCTA CGGGGTCTGA
4381 CGCTCAGTGG AACGAAAACT CACGTTAAGG GATTTTGGTC ATGAGATTAT CAAAAAGGAT
4441 CTTCACCTAG ATCCTTTTAA ATTAAAAATG AAGTTTTAAA TCAATCTAAA GTATATATGA
4501 GTAAACTTGG TCTGACAGTT ACCAATGCTT AATCAGTGAG GCACCTATCT CAGCGATCTG
4561 TCTATTTCGT TCATCCATAG TTGCCTGACT CCCCGTCGTG TAGATAACTA CGATACGGGA

4621 GGGCTTACCA TCTGGCCCCA GTGCTGCAAT GATACCGCGA GACCCACGCT CACCGGCTCC
4681 AGATTTATCA GCAATAAACC AGCCAGCCGG AAGGGCCGAG CGCAGAAGTG GTCCTGCAAC
4741 TTTATCCGCC TCCATCCAGT CTATTAATTG TTGCCGGGAA GCTAGAGTAA GTAGTTCGCC
4801 AGTTAATAGT TTGCGCAACG TTGTTGCCAT TGCTACAGGC ATCGTGGTGT CACGCTCGTC
4861 GTTTGGTATG GCTTCATTCA GCTCCGGTTC CCAACGATCA AGGCGAGTTA CATGATCCCC
4921 CATGTTGTGC AAAAAAGCGG TTAGCTCCTT CGGTCCTCCG ATCGTTGTCA GAAGTAAGTT
4981 GGCCGCAGTG TTATCACTCA TGGTTATGGC AGCACTGCAT AATTCTCTTA CTGTCATGCC
5041 ATCCGTAAGA TGCTTTTCTG TGACTGGTGA GTACTCAACC AAGTCATTCT GAGAATAGTG
5101 TATGCGGCGA CCGAGTTGCT CTTGCCCGGC GTCAATACGG GATAATACCG CGCCACATAG
5161 CAGAACTTTA AAAGTGCTCA TCATTGGAAA ACGTTCTTCG GGGCGAAAAC TCTCAAGGAT
5221 CTTACCGCTG TTGAGATCCA GTTCGATGTA ACCCACTCGT GCACCCAACT GATCTTCAGC
5281 ATCTTTTACT TTCACCAGCG TTTCTGGGTG AGCAAAAACA GGAAGGCAAA ATGCCGCAAA
5341 AAAGGGAATA AGGGCGACAC GGAAATGTTG AATACTCATA CTCTTCCTTT TTCAATATTA
5401 TTGAAGCATT TATCAGGGTT ATTGTCTCAT GAGCGGATAC ATATTTGAAT GTATTTAGAA
5461 AAATAAACAA ATAGGGGTTC CGCGCACATT TCCCCGAAAA GTGCCACCTA AATTGTAAGC
5521 GTTAATATTT TGTTAAAATT CGCGTTAAAT TTTTGTTAAA TCAGCTCATT TTTTAACCAA
5581 TAGGCCGAAA TCGGCAAAAT CCCTTATAAA TCAAAAGAAT AGACCGAGAT AGGGTTGAGT
5641 GTTGTTCCAG TTTGGAACAA GAGTCCACTA TTAAAGAACG TGGACTCCAA CGTCAAAGGG
5701 CGAAAAACCG TCTATCAGGG CGATGGCCCA CTACGTGAAC CATCACCCTA ATCAAGTTTT
5761 TTGGGGTCGA GGTGCCGTAA AGCACTAAAT CGGAACCCTA AAGGGAGCCC CCGATTTAGA
5821 GCTTGACGGG GAAAGCCGGC GAACGTGGCG AGAAAGGAAG GGAAGAAAGC GAAAGGAGCG
5881 GGCGCTAGGG CGCTGGCAAG TGTAGCGGTC ACGCTGCGCG TAACCACCAC ACCCGCCGCG
5941 CTTAATGCGC CGCTACAGGG CGCGTCCCAT TCGCCATTCA GGCTGCGCAA CTGTTGGGAA
6001 GGGCGATCGG TGCGGGCCTC TTCGCTATTA CGCCAGCTGG CGAAAGGGGG ATGTGCTGCA
6061 AGGCGATTAA GTTGGGTAAC GCCAGGGTTT TCCCAGTCAC GACGTTGTAA AACGACGGCC
6121 AGTGAGCGCG CGTAATACGA CTCACTATAG GG
//
